# Indoor air pollution on nurseries and primary schools: impact on childhood asthma – study protocol

**DOI:** 10.1186/1471-2458-12-435

**Published:** 2012-06-13

**Authors:** Sofia I V Sousa, Catarina Ferraz, Maria C M Alvim-Ferraz, Luisa G Vaz, Agostinho J Marques, Fernando G Martins

**Affiliations:** 1 LEPAE, Departamento de Engenharia Química, Faculdade de Engenharia, Universidade do Porto, Rua Dr. Roberto Frias, 4200-465, Porto, Portugal; 2 Departamento de Pediatria (UAG-MC), Hospital de São João, Alameda Prof. Hernâni Monteiro, 4200-319, Porto, Portugal; 3 Departamento de Medicina, Faculdade de Medicina, Universidade do Porto, Alameda Prof. Hernâni Monteiro, 4200-319, Porto, Portugal

**Keywords:** Indoor air, Pollution mixtures, Childhood asthma, Prevalence, Incidence, Exacerbation

## Abstract

**Background:**

Several studies have demonstrated an association between the exposure to indoor air pollution (IAP) and childhood asthma. Evidence is suggesting that several air pollutants may contribute to both exacerbation and development of asthma, but some uncertainty remains concerning the specific causative role of IAP. This paper reports an epidemiologic study aiming to reduce the existing lacks on the association between long-term exposure to pollution mixtures and the development and exacerbation of childhood asthma.

**Methods/design:**

Based on the implementation of the study in 8 nurseries and 8 primary schools, from which, 2 nurseries and 2 primary schools in sites influenced by traffic and other 2 nurseries and 2 primary schools in background sites at urban and rural areas, the study will analyse the exposure to both urban and rural pollution as well as to traffic emissions (some homes of the children will be included in the study). Furthermore, based on the answers to validated questionnaires (as those used in the International Study of Asthma and Allergies in Childhood - ISAAC) filled in by the parents and on medical exams, the study will assess the prevalence, incidence and exacerbation of asthma, thus considering both short and long-term effects. The approximate number of children in the study will never be less than 600, guaranteeing 80% of study power (significant at a 5% level).

**Discussion:**

This study intends to contribute for the understanding of the role of environmental factors, namely indoor air pollution, on asthma considering a risk group of different ages, and for the development of preventive measures, which are considered priority issues by the European Commission, according to the European Environmental Agency and the World Health Organization.

## Background

Until rather recently, the emphasis on air quality evaluation has been centred upon the outdoor environment, namely on studies regarding behaviour, effects and outdoor air pollutants prediction [1-5] and it has been reported that outdoor pollution, specifically nitrogen dioxide (NO_2_), particulate matter with an aerodynamic diameter equal or smaller than 10 μm (PM_10_) and ozone (O_3_), causes an increase on asthma prevalence and also on its severity, having worst effects on children [6-11]. It is now known that IAP likely has equal or even greater impact on children’s health when compared to that of outdoor pollutants. This occurs because time spent indoor is usually higher than time spent outdoor; also, there is a great variety of indoor sources, that include outdoor and specific indoor sources associated with formaldehyde and volatile organic compounds (VOC) emissions, leading frequently to higher concentration than outdoor [12-14]. The World Health Organization (WHO) has assessed the contribution of a range of risk factors to the burden of disease and revealed IAP as the 8th most important risk factor and responsible for 2.7% of the global burden of disease [15]. Exposure to IAP has been linked to a variety of health effects, including respiratory health problems and exacerbation of childhood asthma [16].

Children are highly vulnerable to air pollution effects for a variety of reasons, being considered a risk group [17,18]. Evaluating the risk of developing childhood asthma is one of the four priority issues identified by the European Community, according to the European Union Environment and Health Action Plan [19]. Additionally, prevention of the health effects of poor indoor air quality is needed in all regions of the world [15]. Current evidence on the effectiveness of different interventions is insufficient for providing clear guidance to decision-makers on suitable strategies to reduce the health effects caused by IAP [15].

Accordingly, indoor air quality at schools has attracted increasing public attention in recent years because children spent most of their time at school (8–10 h per day, of which 2–3 h are spent outdoors) [19]. Building materials can release a wide range of pollutants, such as formaldehyde from chipboard and hydrocarbons from paints, cleaners and furnishings [15]. Frequently, pollutants from indoor sources may build up to appreciable levels because of the slowness of air exchange.

Several studies have demonstrated associations between exposure to IAP and exacerbation of childhood asthma [12,20,21]. Nevertheless, uncertainty remains in the causative role of IAP in asthma, but evidence is suggesting that several air pollutants may contribute to both exacerbation and development of asthma.

IAP constitutes a complex case for risk assessment due to a wide number of reasons such as the variety of pollutants, exposure levels, cultural habits, building stock and climate. Also comparison between study areas is a difficult issue because of the different methodologies applied for both pollutant measurements and asthma identification. Generally, IAP studies were performed for specific pollutants and areas, thus not considering all of the most important indoor pollutants and with no possible way to use for comparison with other areas. Additionally, a great uncertainty still remains in the exposure patterns, thus models have been developed to predict the emissions and distribution patterns of pollutants. Such models are essential for the development of indoor exposure and risk assessment [22,23]. However, no general model has yet been well established [24]. Another difficulty remains in the study design; generally, studies investigating the respiratory health effects of IAP on children show either the short or the long-term effects, thus evaluating only prevalence or incidence or exacerbation and usually only in a determined age group [24].

As far as our knowledge goes, only 4 projects were approved in Portugal concerning indoor air quality and health effects on children. These studies were lacking the evaluation of some important indoor air pollutants, each study only evaluated one strict age group, study design was cross-sectional, evaluating only asthma prevalence or only asthma exacerbation and comparison between regions with different air pollution characteristics was not considered [25-28].

INAIRCHILD aims to reduce these lacks, going further on the subject by: i) characterizing in detail the indoor air quality both in nurseries and primary schools, considering all the important indoor air pollutants (carbon monoxide (CO), carbon dioxide (CO_2_), PM_10_, NO_2_, O_3_, VOC, formaldehyde, radon, bacteria, including legionella when necessary) and fungi; ii) understanding and comparing simultaneously the effects of environment on asthma at different age groups (children will have from 1–10 years old); iii) performing a longitudinal study, allowing the evaluation of asthma prevalence, incidence and, if possible, exacerbation; iv) comparing regions of different air pollution characteristics, namely urban and rural areas in terms of indoor pollution and asthma prevalence, incidence and, if possible, exacerbation; and v) contributing to a better supported development of preventive measures.

Globally, the expected results of the project are: i) to identify the pollutants that eventually increase the prevalence and incidence of childhood asthma and exacerbate respiratory symptoms, which will allow developing strategies to reduce the levels of those pollutants aiming the reduction of the incidence rate of childhood asthma; ii) to support directly and indirectly the environmental education, namely through the distribution of flyers, and through the participation of scholar communities (including parents) on the study and on promoting strategies to reduce the levels of indoor pollution and thus the impact on childhood health; and iii) dissemination of the results obtained to the scientific community and the communication of the results to the competent political authorities allowing an eventual revision and adaptation of standards to effectively protect children health.

## Methods/design

**Figure 1:**
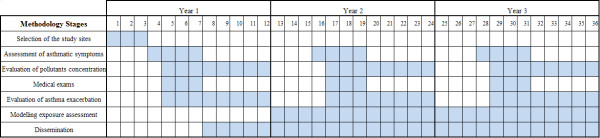
Project timeline.

The study will be performed in 3 years and will include seven main stages as described below. Some of these stages will be performed simultaneously along the 3 years period, as can be seen in Figure 1. The study has been approved by the Ethical Committee of *São João* Hospital.

### Selection of the study sites

At this stage it is expected to select the sites where the study will be performed, including measurement sites indoor and outdoor at nurseries, primary schools and homes.

The sites of study will be selected in the Porto Metropolitan Area (AMP, urban area) and in *Bragança* area (North of Portugal, rural area), by analysing data related to asthmatic children from the central hospital that covers both areas. Data will include information about area of residence, outdoor environment, nursery/primary school identification, social conditions of the children and home facilities.

The study will be implemented in 8 nurseries and 8 primary schools, 4 in AMP and 4 in a rural area (those which accept by written consent to participate in the study). From those, 2 nurseries and 2 primary schools in sites influenced by traffic and other 2 nurseries and 2 primary schools in background sites at both areas. This will be performed to analyse both the influence of urban and rural areas and that of pollutants related with traffic. It is expected to have at least 800 children in each area participating in the study. Considering that the prevalence of childhood asthma in Portugal is around 10%, and that the background prevalence (at a site with low concentrations of air pollutants) is around 3%, the study power will be 95% (significant at a 5% level). However, if the number of children is lower, it will be never below 600 in total, guaranteeing an 80% of power in the conditions described above.

Furthermore, some homes of the children will be included in the study (the number of homes will be calculated through statistical inference, but it will be never less than 10% of the number of children included in the study).

### Assessment of asthmatic symptoms

At this stage it is expected to assess asthmatic symptoms, and gather information about children’s background.

Parents will be asked to sign a participation consent according to the Helsinki Declaration developed by the World Medical Association.

After the selection of study sites, questionnaires validated by medical doctors will be distributed to the parents who agreed to participate in the study, including questions concerning education, social economic status, environmental tobacco smoke, food habits, allergies, amount of time spent indoor and outdoor, home facilities and respiratory health symptoms. Children will be considered asthmatic when dyspnoea and wheezing were simultaneously mentioned in the absence of upper respiratory infections. Children aged 6–10 will perform medical tests even when mentioning one of asthma symptoms: wheeze, dyspnoea or cough in the absence of upper respiratory infections. Due to the difficulty in confirming asthma in children aged 1–5 by medical tests, this group will be assessed only by questionnaires.

The same questionnaires will be distributed again in the following years, in the same nurseries and primary schools, but to groups of the same age as those answering initially, to evaluate asthma incidence.

### Evaluation of pollutant concentrations

At this stage it is expected to: i) determine the most important indoor air pollutants present at nurseries, primary schools and homes of children aged 1 to 10 years old, in two distinct environments of the North of Portugal (urban and rural areas) to evaluate the effects of two different lifestyles; ii) determine the contribution of outdoor pollution in the indoor environment in both urban and rural areas and comparison of background sites and sites influenced by traffic emissions; and iii) assess and compare the indoor sources (namely heating systems, ventilation systems, air conditioning, environmental tobacco smoke, fossil fuels for home cooking) and concentrations of indoor air pollutants identified at those two different environments.

After collecting the questionnaires that will include questions concerning the homes characteristics of the selected children, this task will initiate with the logistic preparation of campaigns for measuring indoor concentrations of CO, CO_2_, PM_10_, NO_2_, O_3_, VOC, formaldehyde, radon, bacteria, including legionella when necessary, and fungi; chemical pollutants will be also be measured outdoor (nitogen oxides (NO_x_), PM_10_, O_3_ and VOC). Measurements will be performed, by standard methods [29] and according to Directive 2008/50/EC, in warm and cold months, thus allowing the evaluation of the pollutants seasonality and its respective effects on the respiratory symptoms of children. Meteorological parameters such as temperature and relative humidity will be also measured continuously both indoor and outdoor (at nurseries/primary schools and at homes).

### Medical exams

At this stage it is expected to evaluate the prevalence and incidence of asthmatic children at the selected sites.

After the assessment of the questionnaires all asthmatic children will be examined for allergies through total Immunoglobulin E (IgE) and depending on the result specific IgE for *Dermatophagoides* spp. and fungi will be tested. Children aged 6–10 will be examined by medical doctors through spirometric tests, to confirm the asthmatic symptoms.

Based on the spirometry results, children will be considered asthmatic when: i) the percentage of the Forced Expiratory Volume in the 1^st^ second (FEV_1_) divide by the Forced vital Capacity (FVC) was less than 80%; or ii) the change in FEV_1_ pre and post medication was equal or higher than 12%; or iii) the change in Forced expiratory Flow over the mid-range of expiration (FEF_25%-75%_) pre and post medication was equal or higher than 35% (according to the American Thoracic Society and European Respiratory Society). These tests will be repeated each year to assess the incidence of childhood asthma at the selected areas.

### Evaluation of asthma exacerbation

At this stage it is expected to evaluate the exacerbation of asthmatic symptoms of asthmatic children at the selected sites.

Questionnaires validated by medical doctors will be distributed to the parents of asthmatic children (confirmed by medical exams), whenever pollution episodes occur, to assess acute respiratory symptoms during episodes and on the following days.

Furthermore, an attempt to identify the amount of sold asthma medicines will be performed at pharmacies near the study sites.

### Modelling exposure assessment

At this stage it is expected to identify pollutants with worst effects in both urban and rural environments.

Models will be developed considering the effects of indoor pollution on the respiratory symptoms of children. The effect of each pollutant will be evaluated after adjusting for potential confounders. Assessment will be performed also aiming the identification of pollutants with significant effects in both urban and rural environments. The models to be used will depend on the results obtained but will be based on the concepts developed by Duan and Ott [30].

### Dissemination

As many times as possible, the results will be communicated to the local communities and responsible entities for the air pollution management thus helping local communities to better understand indoor pollution and its sources and contributing to the development of preventive measures. Scientific community will also be aware through the publication of papers at a national and international level. Additionally, a final conference/seminar will be organized for communication of the results.

## Discussion

Based on the above described the expected results are the following:

i. Identification of the pollutants that eventually increase the prevalence and incidence of childhood asthma and exacerbate respiratory symptoms. This will allow developing strategies to reduce the levels of those pollutants aiming the reduction of the incidence rate of childhood asthma.

ii. Support directly and indirectly the environmental education, namely through the distribution of flyers, and through the participation of scholar communities (including parents) on the results obtained and on the strategies to reduce the levels of indoor pollution and thus the impact on childhood health.

iii. Publication in international and national journals and the communication in international and national conferences that will allow reporting the results obtained to the scientific community. Additionally, the communication of the results to the competent political authorities that will allow the eventual revision and adaptation of standards to the effective protection of children health.

iv. To find out the most important sources of indoor air pollution in nurseries and primary schools, depending not only on the indoor conditions but also on the outdoor environment (urban or rural influenced by traffic or background).

v. To understand the seasonality of the specified pollutants and the influence of meteorological parameters in each one of them and its influence on the asthmatic symptoms, according to children exposure.

vi. To assess the prevalence and incidence of asthma at an urban and at a rural area and associating it with the exposure of children to indoor and outdoor pollution considering the confounding effects.

vii. To understand if there are synergistic effects between pollutants and between them and meteorological parameters that can affect the development and exacerbation of childhood asthma.

## Abbreviations

FEV1, Forced Expiratory Volume in the 1st second; FVC, Forced vital Capacity; FEF25%-75%, Forced expiratory Flow over the mid-range of expiration; IgE, Immunoglobulin E; IAP, Indoor Air Pollution; CO, Carbon monoxide; CO2, Carbon dioxide; NOx, Nitogen oxides; NO2, Nitrogen dioxide; PM10, Particulate matter with an aerodynamic diameter equal or smaller than 10 μm; O3, Ozone; VOC, Volatile Organic Compounds; WHO, World Health Organisation; AMP, Porto Metropolitan Area.

## Competing interests

The authors declare that they have no competing interests.

## Authors’ contributions

SIVS is the principal investigator, conceived the study, led the study design and coordination and drafted the manuscript. CF, LGV and AJM were responsible for the design of the health data collection and will perform the medical exams and the respective analysis; they also critically revised the manuscript. MCMAF and FGM contributed to the design of the study and critically revised the manuscript. All authors read and approved the final manuscript.

## Pre-publication history

The pre-publication history for this paper can be accessed here:

http://www.biomedcentral.com/1471-2458/12/435/prepub
